# Anticholinergic and benzodiazepine medication use and risk of incident dementia: a UK cohort study

**DOI:** 10.1186/s12877-019-1280-2

**Published:** 2019-10-21

**Authors:** Carlota M. Grossi, Kathryn Richardson, Chris Fox, Ian Maidment, Nicholas Steel, Yoon K. Loke, Antony Arthur, Phyo Kyaw Myint, Noll Campbell, Malaz Boustani, Louise Robinson, Carol Brayne, Fiona E. Matthews, George M. Savva

**Affiliations:** 1 School of Health Sciences, University of East Anglia, Norwich, NR4 7TJ UK; 2 Norwich Medical School, University of East Anglia, Norwich, NR4 7TJ UK; 3 School of Life and Health Sciences, Aston University, Birmingham, B4 7ET UK; 4 Institute of Applied Health Sciences, University of Aberdeen, Aberdeen, AB25 2ZD UK; 5 Department of Pharmacy Practice, College of Pharmacy, Purdue University, West Lafayette, USA; 6 Indiana University School of Medicine, Indianapolis, Indiana USA; 7 Institute of Health and Society/Institute for Ageing, Newcastle University, Newcastle, NE4 5PL UK; 8 Cambridge Institute of Public Health, University of Cambridge, Cambridge, CB2 0SR UK; 9 Quadram Institute Bioscience, Norwich, Norfolk UK

**Keywords:** Alzheimer disease, Cognition, Dementia, Cohort study, Benzodiazepines, Cholinergic antagonists

## Abstract

**Background:**

Studies suggest that anticholinergic medication or benzodiazepine use could increase dementia risk. We tested this hypothesis using data from a UK cohort study.

**Methods:**

We used data from the baseline (Y0), 2-year (Y2) and 10-year (Y10) waves of the Medical Research Council Cognitive Function and Ageing Study. Participants without dementia at Y2 were included (*n* = 8216). Use of benzodiazepines (including nonbenzodiazepine Z-drugs), anticholinergics with score 3 (ACB3) and anticholinergics with score 1 or 2 (ACB12) according to the Anticholinergic Cognitive Burden scale were coded as ever use (use at Y0 or Y2), recurrent use (Y0 and Y2), new use (Y2, but not Y0) or discontinued use (Y0, but not Y2). The outcome was incident dementia by Y10. Incidence rate ratios (IRR) were estimated using Poisson regression adjusted for potential confounders. Pre-planned subgroup analyses were conducted by age, sex and Y2 Mini-Mental State Examination (MMSE) score.

**Results:**

Dementia incidence was 9.3% (*N* = 220 cases) between Y2 and Y10. The adjusted IRRs (95%CI) of developing dementia were 1.06 (0.72, 1.60), 1.28 (0.82, 2.00) and 0.89 (0.68, 1.17) for benzodiazepines, ACB3 and ACB12 ever-users compared with non-users. For recurrent users the respective IRRs were 1.30 (0.79, 2.14), 1.68 (1.00, 2.82) and 0.95 (0.71, 1.28). ACB3 ever-use was associated with dementia among those with Y2 MMSE> 25 (IRR = 2.28 [1.32–3.92]), but not if Y2 MMSE≤25 (IRR = 0.94 [0.51–1.73]).

**Conclusions:**

Neither benzodiazepines nor ACB12 medications were associated with dementia. Recurrent use of ACB3 anticholinergics was associated with dementia, particularly in those with good baseline cognitive function. The long-term prescribing of anticholinergics should be avoided in older people.

## Background

Dementia prevention is a public health priority. No disease modifying treatment for dementia exists, but dementia risk and progression can be modified by changing exposure to risk factors affecting any aspect of long-term brain health [1]. Identifying such risk factors is important for dementia prevention and cognitive health.

Long-term use of several classes of medications have been suggested to increase future dementia risk. Medications with anticholinergic activity (henceforth anticholinergics), benzodiazepines and related non-benzodiazepine derivatives have come under particular scrutiny owing to their well-known short-term cognitive effects [2] and the high prevalence of their long-term use among middle aged and older people [3, 4].

Anticholinergics are successfully used in the treatment of many conditions such as urinary incontinence, Parkinson’s disease, depression, and epilepsy. Anticholinergics can adversely affect cognition [2]; guidelines suggest they are to be avoided among frail older people [5] or those with dementia [6]. Over the past decade, prolonged exposure to anticholinergics has been linked to long term cognitive decline or dementia [7–12]. Many medicines beyond those typically regarded as anticholinergics may have mild anticholinergic effects and it has been suggested that the cumulative long term use of many such medications may increase dementia risk [11]. Depending on their definition, anticholinergic medications are used by 10–50% of the middle aged and older population at any time [13, 14].

Benzodiazepines and non-benzodiazepine derivatives are primarily used to treat anxiety or insomnia. Short term cognitive effects due to their sedating action are well recognised. Although long-term use is not recommended many people use regularly benzodiazepines and related medicines for years or decades [3]. Estimates of the effect of benzodiazepine use on long term cognitive decline and dementia have been mixed [15–22].

For both benzodiazepines and anticholinergics, several methodological biases exist in the published studies including first the lack of longitudinal observational window with a clear baseline measurement of cognitive and functional status of the population at risk; second the absence of gold standard measurement of the dementia incidence; third, no precise baseline measurement of the exposure variable (benzodiazepine and anticholinergic use); and finally the limitation of observational studies in resolving protopathic bias, whereby medication use might be prescribed for symptoms at the very early stages of dementia, inducing an association between medication use and later dementia diagnosis.

We have previously demonstrated association between cognitive decline and anticholinergic use between baseline and 2-year follow-up assessments of the Medical Research Council Cognitive Function and Ageing Study (MRC CFAS) [9]. Here we extend this analysis to examine dementia incidence at 10 year follow-up, with respect to patterns of anticholinergic and benzodiazepine use at the baseline and 2-year follow-up assessments.

## Methods

### Setting

The MRC CFAS is a population based, prospective, multicentre cohort study in England and Wales specifically designed to estimate the prevalence, risk factors and course of dementia. The study design has been described elsewhere [23]; (see also www.cfas.ac.uk for full details).

In brief, 13,004 participants, age 65 and older, from Cambridgeshire, Gwynedd, Newcastle, Nottingham and Oxford, were recruited with baseline interviews (Y0) conducted between 1991 and 1993. All individuals still alive and traceable were invited to be re-interviewed at two years (Y2) and 10 years (Y10) after baseline. At each wave, participants were questioned about sociodemographic factors, lifestyle, physical and mental health (including self-reported insomnia, measures of anxiety and depression) and completed a cognitive battery and in-home medication inventory. For the present analysis, we included all those who participated at Y2 with no study diagnosis of dementia at Y0 or at Y2, and measured incident dementia as an outcome at Y10.

### Outcome assessment

**Fig. 1:**
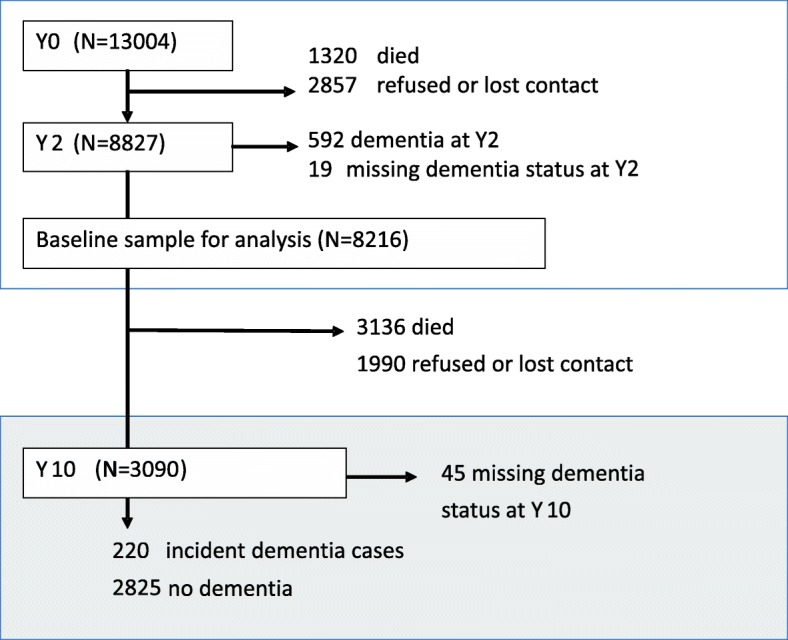
Flow of participants included in the current analysis through the MRC Cognitive Function and Ageing Study. See www.cfas.ac.uk for the full design of the Cognitive Function and Ageing Studies

At Y0 and Y2 the study diagnosis of dementia was made using a two-phase process (Fig. 1). An initial screening interview was administered to all participants. A stratified subsample of 20%, including all of those with cognitive impairment, but also including healthy participants then underwent a thorough assessment using the Automated Geriatric Examination for Computer Assisted Taxonomy (AGECAT) algorithm to make a study diagnosis of dementia [23–26]. AGECAT produces a score of between 0 and 5. Dementia was defined as AGECAT scores ≥3 which is equivalent to dementia as diagnosed by DSM-III-R [24]. All surviving participants underwent the full assessment at Y10.

For those who underwent a screen interview but were not selected to undergo the assessment we imputed the Y2 dementia status based on cognitive screen scores (using a multiple imputation). This procedure identified that there were possibly a small number of cases of dementia among the screen-only sample, but these were only very rarely seen among those surviving Y10 sample. Hence our primary analysis assumed no prevalent dementia cases among the Y2 screen-only participants; participants who were imputed to have dementia at baseline were excluded in a sensitivity analysis.

### Medication exposures

During each interview participants were asked to provide details of all medication currently being used, either prescribed or bought over-the-counter. These were recorded using UK National Health Service Read codes. Packaging was checked and proxy respondents supplied medication information if participants were unable to do so. Previous studies in older population have demonstrated self-reported medication data gathered in this way to be mostly in moderate-good agreement with prescription data records [27].

All medications were coded according to the Anticholinergic Cognitive Burden (ACB) scale [28]. In summary, medications with serum anticholinergic activity or in vitro affinity to muscarinic receptors but with no known clinically relevant negative cognitive effects are scored 1 on the scale, while drugs with established and clinically relevant anticholinergic effects are scored 2 based on blood-brain penetration and 3 if also have reported associations with delirium. All other drugs are scored 0. Very few medications were classed as having an ACB score of 2, so we created binary exposure variables for ACB12 (use of any medications scoring 1 or 2) and ACB3 (use of any medications scoring 3). A total ACB sum score, and a variable corresponding to the sum of ACB12 drugs only was also created. Each of these exposures was determined independently at Y0 and Y2.

Similarly for benzodiazepines, a binary variable (BZD) corresponding to taking any benzodiazepine or non-benzodiazepine derivative (hypnotics such as zopiclone also known as Z-drugs) was created at both Y0 and Y2.

For each group (BZD, ACB12 and ACB3) participants were then classified as being an ‘ever-user’ (if there was any use at Y0 or Y2), and then sub-classified as a ‘recurrent user’ (use at Y0 and Y2), new user (only at Y2), or as a discontinuing user (only at Y0).

### Covariates

We selected covariates that might have a confounding effect between the use of benzodiazepines or anticholinergics and incident dementia. We included demographic variables of sex, age, education (≤ 9 years, ≥ 10 years), social class (measured by prior occupation as manual vs non manual), centre of recruitment, and study arm (screen or assessment), variables that are indicators for ACB3 or BZD use, early symptoms of dementia or known to be associated with dementia (reporting having suffered stroke, Parkinson disease, epilepsy, sleep problems, anxiety, depression or being diagnosed depression at either Y0 or Y2, as binary variables), self- reported health (excellent/good; fair/poor) at Y2 and cognition related variables.

Pre-existing cognitive impairment and ongoing cognitive decline are the most important potentially confounding factors, these were measured by the Mini-Mental State Examination (MMSE) at Y2 (≤25, > 25), the decrease in MMSE scores between Y0 and Y2 (< 1, 1, 2,  ≥ 3 points), the MMSE orientation sub-score at Y2 (< 9, 9/10) and self-perceived change in memory function between recruitment and 2 years (No change or better vs worse). Disability at Y2 was classified using the Townsend disability scale as either no impairment, any impairment in instrumental activities of daily living or any impairment in basic activities of daily living [29].

### Statistical analyses

Separate univariable Poisson regression models with Huber-White robust standard errors were used to estimate incidence rate ratios (IRR) for the association between each potential predictor variable and incident dementia at Y10 [30]. 95% confidence intervals are reported for all estimates.

In multivariable analysis we additionally included each of the three ever-use variables (where they were not the exposure of interest) and the demographic, health and cognition related variables mentioned above.

We carried out pre-planned stratified analyses of the main ‘ever-use’ models by year of birth (≤1919 vs 1920 onwards), sex and MMSE score at Y2 (> 25, ≤25). The threshold for cognitive function and age were chosen as they reflect the stratification of the original CFAS study sampling.

As expected in this population there was substantial loss to follow-up between Y2 and Y10 caused by drop out and death. Inverse probability weights were used to adjust for non-response at Y10 and loss of contact between Y2 and Y10 or refusal to participate at Y10, conditional on having survived. These weights were calculated using a logistic regression model for being successfully re-assessed at Y10 (conditional on surviving to Y10) including the main effects of all exposures (BZD, ACB12 and ACB3), covariates and the interactions between exposures and sex and MMSE at Y2.

STATA 14.1 was used for all analysis.

### Sensitivity analyses

We carried out three sensitivity analyses to test the impact of modelling assumptions or analytical choices on our results. First, we excluded potentially mediating or colliding variables: MMSE at Y2, change in MMSE (Y0 to Y2), MMSE orientation sub-score at Y2, disability, and arm of the study. Second, we used multiple imputation to identify screen-only participants with dementia at baseline based on their demographic information and cognitive scores as described above, and excluded them from each imputed analysis. Finally, we took into account the possibility that higher mortality rates among older people taking anticholinergics or benzodiazepines and related medications might suppress our estimates of dementia incidence in this group via inverse probability weights calculated using on the probability of death or drop-out (rather than drop-out alone) between Y2 and Y10 based on baseline factors.

## Results

See Fig. 1 for participant flow through the study. From the 13,004 participants recruited to MRC CFAS at Y0, 8216 were interviewed at Y2, did not have dementia or unknown dementia status at Y2 and so form the baseline sample for our incidence analysis. Of these, 3136 died and 1990 were lost to follow up before Y10. At Y10, we excluded a further 5 participants classified as having dementia at Y0 but not Y10 and 45 with unknown dementia status at Y10, leaving 220 people with incident dementia and 2825 people without incident dementia included in the study.

Table 1 shows participant characteristics stratified by follow-up status. Those who developed dementia by Y10 were older, had lower cognitive function at Y2 (mean MMSE 24 vs 27), more disability (ADL-IADL 22% vs 7%), fewer years of education (≥ 10 years 29% vs 44%) and were substantially more likely to report worsening memory from recruitment to 2-year follow up (49% vs 27%) and poorer health (32% vs 21%).

**Table 1:** Participant characteristics stratified by Y10 follow-up status and dementia outcome

Characteristic	Dementia at Y10 (n = 220)	No dementia at Y10 (*n* = 2825)	Lost to follow up between Y2 and Y10 (*n* = 1990)	Died between Y2 and Y10 (*n* = 3136)
Female	163 (77.3)	1675 (61.2)	1315 (66.1)	1630 (52.0)
Mean age (SD)	77.1 (7.0)	72.0 (10.0)	73.8 (6.1)	76.9 (6.7)
Educated for ≥ 10 years	68 (29.3)	1311 (43.9)	754 (37.9)	1127 (35.9)
Manual occupation	132 (61.7)	1359 (50.1)	1088 (54.7)	1751 (55.8)
CFAS assessment arm	84 (51.0)	594 (27.4)	814 (40.9)	999 (31.9)
Y2 MMSE				
≤ 21	30 (22.6)	57 (3.5)	190 (9.6)	381 (12.2)
22–25	76 (38.4)	432 (20.0)	552 (27.7)	845 (27)
26–30	114 (39.0)	2336 (76.5)	1196 (60.1)	1847 (58.9)
Decline in MMSE between Y0 and Y2				
No decline / improvement	100 (41.1)	1592 (55.0)	988 (49.6)	1444 (46.0)
1 point	34 (13.1)	529 (17.6)	302 (15.2)	487 (15.5)
2 points	34 (13.6)	350 (11.8)	220 (11.1)	364 (11.6)
≥ 3 points	50 (29.0)	326 (14.2)	402 (20.2)	729 (23.2)
Disability				
None	121 (47.1)	2336 (80.0)	1386 (69.6)	1562 (49.8)
IADL impairment	65 (30.2)	350 (13.1)	331 (16.6)	672 (21.4)
ADL impairment / unclassified	34 (22.7)	139 (7.0)	273 (13.7)	902 (28.8)
Self-reported memory decline (Y0 to Y2)	110 (48.7)	774 (27.4)	592 (29.7)	1152 (36.7)
Fair/poor self-reported health	66 (31.7)	529 (21.2)	523 (26.3)	1174 (37.4)
Comorbidity^a^				
Sleep disturbance	56 (26.5)	606 (22.6)	507 (25.5)	902 (28.8)
Diagnosed depression	22 (11.0)	309 (11.3)	216 (10.9)	290 (9.2)
Consulted GP for depression	31 (15.8)	388 (14.4)	282 (14.2)	387 (12.3)
Consulted GP for anxiety	28 (11.8)	242 (8.5)	186 (9.3)	228 (7.3)
BZD use^b^				
None	195 (86.6)	2623 (91.7)	1763 (88.6)	2726 (86.9)
Any ^e^	25 (13.5)	202 (8.3)	222 (11.2)	391 (12.5)
New ^f^	5 (2.2)	43 (1.9)	49 (2.5)	92 (2.9)
Discontinuing ^g^	6 (3.2)	51 (2.0)	57 (2.9)	95 (3.0)
Recurrent ^h^	14 (8.1)	108 (4.4)	116 (5.8)	204 (6.5)
ACB3 use^c^				
None	198 (89.8)	2677 (94.1)	1842 (92.6)	2831 (90.3)
Any ^e^	22 (10.2)	148 (5.9)	143 (7.2)	286 (9.1)
New ^f^	5 (1.8)	55 (2.2)	58 (2.9)	112 (3.6)
Discontinuing ^g^	5 (3.6)	35 (1.5)	43 (2.2)	78 (2.5)
Recurrent ^h^	12 (4.9)	58 (2.2)	42 (2.1)	96 (3.1)
ACB12 use^d^				
None	85 (41.0)	1353 (47.3)	908 (45.6)	972 (31)
Any ^e^	135 (59.0)	1472 (52.7)	1077 (54.1)	2145 (68.4)
New ^f^	34 (16.0)	321 (11.4)	210 (10.6)	419 (13.4)
Discontinuing ^g^	11 (4.5)	209 (7.9)	175 (8.8)	327 (10.4)
Recurrent ^h^	90 (38.5)	942 (33.5)	692 (34.8)	1399 (44.6)

Number of participants (percentages) given unless specified otherwise. Percentages of participants at Y10 are weighted for attrition due to non-response at Y10 and loss of contact between Y2 and Y10

Abbreviations: *CFAS* Cognitive Function and Ageing Study, *SD* standard deviation, *MMSE* Mini-Mental State Examination, *IADL* Instrumental Activities of Daily Living, *ADL* Activities of Daily Living, *GP* General Practitioner

^a^Any record of specific comorbidity at the Y0 or Y2 assessment

^b^Use of benzodiazepines or Z-drugs

^c^Use of drugs scoring 3 on the Anticholinergic Cognitive Burden scale

^d^Use of drugs scoring 1 or 2 on the Anticholinergic Cognitive Burden scale

^e-h^Drug use categories are

None: no use at Y0 or Y2;

Any: Use at Y0 or Y2;

New: Use at Y2 but not Y0

Discontinuing: Use at Y0 but not Y2

### Medication use

A breakdown of baseline exposures by 10-year follow-up status is shown in Table 1. Full details of drug use are in Additional file 1. Among those surviving to 10 years, 7.5% reported ever use of a BZD (short-acting 4.2%, long-acting 3.7%). Hypnotic BZD were used by 5.9% with 1.9% using anxiolytics. The most commonly reported BZDs were Temazepam (47% of BZDs reported), Nitrazepam (30%) and Diazepam (15%). Non-benzodiazepine Z-drug use was rare in this cohort (prevalence of 0.4%).

Use of ACB3 at baseline or 2-year follow-up was reported by 5.6% of the surviving sample; 2.3% were recurrent users. The majority of ACB3 drugs were antidepressants (3.8% of the surviving sample; corresponding to 69% of ACB3 medications), urologicals (0.7% reported ever use among the sample), gastrointestinal (0.6%), antipsychotics (0.5%), antihistamines (0.3%) and Parkinsonian drugs (0.1%). The most common ever-use ACB3 medications were the antidepressants: amitriptyline (22% of ACB3) and dosulepin (22% and of ACB3).

In total, 53% of the surviving sample reported ACB1 or ACB2 at baseline or 2-year follow-up, with 34% reporting ACB1 or ACB2 use at both waves.

Although Y10 medication is not considered an exposure in our study, we compared Y10 to Y0 and Y2 medication to understand to what extent medication use was likely to have continued in the overall study sample. Medication use at Y10 was highly correlated with use at Y0 and Y2 (see Additional file 2) with around 60% of ‘recurrent’ users at Y0 and Y2 reported use of each class at Y10. This suggests that in many cases use at Y0 and Y2 is likely to reflect repeated use during the follow-up period as opposed to being one-off exposures.

### Dementia incidence

Table 2 describes incident dementia in our sample as well as the unadjusted and adjusted incidence rate ratios (aIRR). After weighting, 9.5% (*N* = 220) of participants had a study diagnosis of dementia at Y10; 14.5, 15.4 and 10.5% for BZD, ACB3, ACB12 ever-users and 16.0, 18.6 and 10.7% for recurrent users, respectively.

**Table 2:** Attrition-weighted unadjusted and multivariable adjusted incidence rate ratios for the association between benzodiazepine and anticholinergic medication use and incident dementia

Exposure and pattern of use	Dementia incidence			Unadjusted IRR (95% CI)	Adjusted^b^ IRR (95% CI)
	Cases	Total	%^a^		
BZD use					
None	195	2819	9.0	1 (Ref.)	1 (Ref.)
Any	25	227	14.5	1.61 (1.06,2.46)	1.06 (0.72,1.60)
New	5	48	11.1	1.23 (0.51,2.96)	0.65 (0.27,1.60)
Discontinuing	6	57	14.1	1.57 (0.74,3.35)	1.06 (0.53,2.14)
Recurrent	14	122	16.0	1.78 (1.02,3.12)	1.30 (0.79,2.14)
ACB3 use					
None	198	2876	9.1	1 (Ref.)	1 (Ref.)
Any	22	170	15.4	1.70 (1.09,2.65)	1.28 (0.82,2.00)
New	5	60	7.9	0.88 (0.37,2.09)	0.87 (0.34,2.22)
Discontinuing	5	40	20.1	2.22 (0.96,5.14)	1.19 (0.53,2.68)
Recurrent	12	70	18.6	2.05 (1.18,3.56)	1.68 (1.00,2.82)
ACB3 subclass					
Not antidepressants	6	53	13.6	1.50 (0.68,3.32)	1.74 (0.84,3.62)
Antidepressants	16	117	16.1	1.78 (1.06,2.98)	1.16 (0.69,1.94)
ACB1 or ACB2 use					
None	85	1438	8.3	1 (Ref.)	1 (Ref.)
Any	135	1609	10.5	1.26 (0.95,1.67)	0.89 (0.68,1.17)
New	34	355	12.8	1.54 (1.02,2.33)	1.14 (0.79,1.63)
Discontinuing	11	220	5.6	0.68 (0.36,1.27)	0.36 (0.19,0.69)
Recurrent	90	1033	10.7	1.29 (0.95,1.75)	0.95 (0.71,1.28)
ACB sum score					
Total ACB score (per point)				1.07 (1.03,1.13)	1.00 (0.94,1.06)
ACB12 score (per point)				1.06 (1.00,1.13)	0.97 (0.90,1.04)
ACB3 score (per point)				1.10 (1.02,1.19)	1.06 (0.98, 1.15)

Abbreviations: *IRR* Attrition-weighted unadjusted incidence rate ratio, *aIRR* Attrition-weighted adjusted incidence rate ratio, *CI* confidence-interval, *ACB* Anticholinergic Cognitive Burden, *ACB1* use of a medicine with an ACB score of 1. Scores correspond to possibly anticholinergic (score 1) probably anticholinergic (score 2) definitely anticholinergic (score 3)

^a^% represents weighted incidence

^b^Adjusted for sex, age, education (≤ 9 years, ≥ 10 years), social class (manual vs non manual), residential accommodation, centre of recruitment, study arm (screen or assessment), health conditions at Y0 or Y2 (stroke, Parkinson disease, epilepsy, sleep problems, anxiety, depression), self- reported health (excellent/good; fair/poor) at Y2, Disability at Y2 (no impairment, impairment in instrumental activities of daily living, or impairment in basic activities of daily living), Mini-Mental State Examination (MMSE) at Y2 (≤25, > 25), MMSE orientation sub-score at Y2 (< 9, 9/10), decrease in MMSE score between Y0 and Y2 (< 1, 1, 2,  ≥ 3 points), and self-perceived change in memory function between Y0 and Y2 (No change or better vs worse)

Adjusted IRRs for dementia at Y10 were 1.06 (95%CI 0.72, 1.60) for any BZD use, 1.28 (95% CI 0.82, 2.00) for any ACB3 and 0.89 (95%CI 0.68 1.17) for any ACB12 use. Recurrent use was associated with IRRs of 1.30 (95%CI 0.79, 2.14) for BZD, 1.68 (95%CI 1.00, 2.82) for ACB3 and 0.95 (95%CI 0.71, 1.28) for ACB12.

There was no evidence for an increase in dementia risk with increasing total ACB score at each wave, or with the number of ACB1 or ACB2 medications used. No significant association was found between dementia and ever-use of short or medium-acting, long-acting, hypnotic or anxiolytic BZDs, or for anti-depressant or ‘other’ anticholinergics although numbers in these subgroups were small (results not shown).

### Stratified analysis

Stratified analyses are shown in Table 3. The effect of ACB3 was restricted to those with good baseline cognitive function (ever-users aIRR: 2.28, 95%CI 1.32, 3.92), whereas no such association was seen among the group with impaired cognition (ever-users aIRR: 0.94, 95% CI: 0.51–1.73). Those with poor cognitive function (MMSE ≤25 at Y2) had a dementia incidence rate of around 21% irrespective of anticholinergic use (21.3%; 97 of 500 among never-users vs 21.8%; 9 of 46 for ever-users), while for those with good cognitive function (MMSE> 25 at Y2) the Y10 dementia incidence rate was 11.1% (13 of 124) for ACB3 ever-users and 4.7% (101 of 2326) for never-users (Additional file 3). This is supported by a statistically significant interaction effect (*p* = 0.02). No other significant subgroup differences were found.

**Table 3:** Attrition-weighted adjusted incidence rate ratios for benzodiazepine and anticholinergic medication use and incident dementia, stratified by cognition, sex and age

	Incidence rate ratio (95% confidence interval) by exposure		
Subgroup	Any Benzodiazepines	Any ACB3	Any ACB12
MMSE at Y2 > 25	0.72 (0.35,1.50)	2.28 (1.32,3.92)*	0.99 (0.68,1.43)
MMSE at Y2 ≤ 25	1.23 (0.74,2.06)	0.94 (0.51,1.73)	0.78 (0.54,1.12)
Male	0.29 (0.06,1.31)	2.06 (0.78,5.46)	1.11 (0.66,1.89)
Female	1.17 (0.77,1.78)	1.24 (0.77,2.01)	0.85 (0.63,1.16)
Younger (born 1920–1929)	1.31 (0.52,3.27)	1.16 (0.45,3.01)	1.57 (0.82,3.00)
Older (born before 1920)	1.06 (0.69,1.61)	1.27 (0.78,2.09)	0.77 (0.58,1.03)

*p < 0.01

Abbreviations: *MMSE* Mini-Mental State Examination, *ACB* Anticholinergic Cognitive Burden, *ACB12* use of a medicine with an ACB score of 1 or 2. Scores correspond to possibly anticholinergic (score 1), probably anticholinergic (score 2) and definitely anticholinergic (score 3). Number of observations and adjusted percentage in each group are reported in Additional file 3

### Sensitivity analyses

Results from the sensitivity analyses are shown in Additional file 4. No changes were seen after removing imputed possible dementia cases at baseline or 2-year follow-up. However, after excluding baseline disability and cognition related variables from multivariable regression there was an increase in the effects of any ACB3 use and recurrent use with aIRRs 1.55 (95%CI 1.04, 2.32) and 2.02 (95%CI 1.21, 3.39), respectively. No main changes were observed when using weights to adjust for mortality or after carrying out a competing risk analysis (results not shown). In analysis stratified by cognitive score, there is no change to main findings in sensitivity analysis; for example when using inverse probability weights to adjust for attrition by death or other loss to follow up the association between baseline ACB3 use and incident dementia among those with MMSE> 25 at Y2 is aIRR = 2.24 (95% CI: 1.24–4.06) compared to IRR = 1.01 (0.55–1.87) among those with Y2 MMSE< 25.

## Discussion

In a cohort study with 10-year follow-up we did not find any evidence of an increase in risk of dementia associated with the use benzodiazepines or anticholinergics scoring ACB1 or ACB2. We did find a statistically significant increase in dementia risk among recurrent users of ACB3 anticholinergics and also an association between ACB3 anticholinergics use and dementia risk among the subgroup with good baseline cognitive function, suggesting that effects might more apparent in different subgroups of the older population.

### Benzodiazepines

Previous studies on the effect of benzodiazepines have been inconsistent, with some large and apparently high quality studies showing a clear effect of benzodiazepine use on dementia incidence [16–18, 20, 21], but others finding no effect [15, 19, 22]. There is no readily apparent difference between these studies in design that explains this inconsistency, although possible explanations include selection biases into electronic health record databases, differing methods of ascertaining benzodiazepine use, such as duration, dose and chronicity and the measurement of dementia outcome [15], or the differing profile of benzodiazepine use [31], population characteristics across studies or the manner in which each study was able to control for covariates. There was insufficient use of Z-drugs among our cohort to draw any conclusions regarding their effects on dementia incidence.

### Strong anticholinergics

Our estimate of the effect of ACB3 anticholinergics on dementia incidence was not statistically significant, but is consistent with recent effect estimates from analyses of electronic medical records [7, 32]. However, in planned subgroup analyses we observed a borderline significant increased dementia risk in recurrent users of ACB3 anticholinergics, defined as those participants who reported anticholinergic use at both baseline and two-year follow-up, more likely to reflect a longer term or continuous anticholinergic load. This is consistent with the hypothesis that long-term as opposed to one-off use is needed to increase dementia risk.

Consistent with our work, previous studies have consistently reported associations between anticholinergic use and dementia incidence, with a greater effect seen among prevalent (as opposed to new users) or long-term recurrent users, with some studies reporting a dose effect with increasing risk at higher doses [7, 32]. New use or short term use has consistently not been associated with risk of developing dementia [8]. Similar results have been observed for studies focussing on cognitive change instead of dementia or MCI outcomes and in neuropathology studies [33, 34].

We stratified our analysis by baseline cognitive function to test the hypothesis that the effect is only seen among people with an existing cognitive impairment, reflecting possible protopathic bias. In fact the reverse was observed, the effect was restricted to those with good baseline cognitive function. It is possible that this reflects increased attrition among the more cognitively frail using anticholinergics, however this finding is not affected by using a weight that corrects for attrition due to death, and in any case this results demonstrates that the increase in dementia incidence associated with anticholinergics is not restricted to those with existing cognitive impairment or those with incipent dementia.

Anticholinergics represent a broad class of medications that act on different systems, and it is possible that different anticholinergics have different long term effects on brain health [12]. Disaggregation of anticholinergic classes may also help to identify possible confounding by indication or protopathic bias. Our study suggests that anticholinergics other than antidepressants have a stronger link with incident dementia than do anticholinergic antidepressants after adjustment for confounding factors, but owing to small numbers estimates of the effects of subclasses are very imprecise [7, 12].

### Anticholinergics with score of 1 or 2

While ACB3 anticholinergics are used by only 3–5% of the older population at any time, up to 50% are using one or more of the much wider group that are considered ‘possibly’ anticholinergic (score of 1), and any effect of these medications on dementia incidence would have a great public health significance [9]. Our finding that the number of ACB12 anticholinergics used is not associated with future incident dementia agrees with our previous analysis of cognitive change between baseline and 2 years [9] and previous studies that have considered these groups separately [12, 35, 36]. The number of medications classified as ACB2 is very small and this effect estimate is largely dominated by the effect of medications classified ACB1. Findings from the Baltimore Longitudinal Study of Ageing suggest an increase in the risk of ‘Alzheimer’s disease or MCI’ with increasing use of ‘possible’ anticholinergics, with an associated increase in cortical atrophy, although there was no effect of definite anticholinergic (score of 3) use suggesting that anticholinergic properties of these drugs may not underlie the effect [37].

### Strengths and limitations

Our study has several important strengths and limitations. By using the first two waves of MRC CFAS (years 0 and 2) as the baseline and dementia at 10-year follow-up as the outcome we could identify the long-term effect of different patterns of uses of medications in a population-representative cohort. We did not measure medication use or dementia diagnoses occurring between assessments, or the diagnoses for those who dropped out before Y10. Although the high concordance between medications used at Y0, Y2 and Y10 suggests that use may have been continual during the follow up period in many cases, we have no direct evidence for this. Medication use was based on self-report and adherence was not formally assessed; although there is no gold standard method for measuring adherence to medication [38]. Dementia was measured using a validated algorithm, and thus any bias due to outcome ascertainment is reduced compared to studies relying on a recorded diagnosis dementia which will significantly under represent true dementia incidence [39].

Despite the large sample size of MRC CFAS (*n* = 13,004), the numbers using benzodiazepines or anticholinergics with score ACB3 during the first two waves and developing incident dementia by Y10 are relatively small. Estimating effects for subgroups is difficult. Attrition over 8 years was typical of that seen in comparable studies of ageing, and we applied inverse probability weighting based on exposures and baseline cognitive scores to adjust for differential drop-out. Use of inverse probability weights assumes that loss to follow-up or death was not differential with respect to unmeasured confounders or to the outcome. Our findings might be biased if the interaction between medication use and dementia has a specific association with drop-out that could not be attributed to either factor alone or the interaction between exposure and pre-existing cognitive impairment.

We controlled for many relevant potential confounders, in particular for many of the indications for anticholinergics and benzodiazepines. We could not control for urinary incontinence or obesity as this was not routinely recorded, however the anticholinergic urologicals were rarely used among this cohort. Mental health disorders apart from depression and anxiety were also not routinely recorded. Adjusting for recent cognitive decline and observing the effect among those with good cognitive function at Y2 helps to exclude the possibility of protopathic bias due to reverse causation.

## Conclusions

We found no evidence that benzodiazepines are associated with dementia incidence but we cannot rule out an effect as the number of benzodiazepine users in our study was relatively small. Consistent with previous studies we found an increase in dementia incidence associated with the recurrent use of anticholinergics with an ACB score of 3, particularly among those with good baseline cognitive function. This should be treated with caution owing to small sample size but when considered alongside the growing body of evidence from cohort studies and administrative data sources suggests that at least some anticholinergic medications could increase the risk of future dementia. The prevalence of anticholinergic medication use remains high among middle aged and older people, making this a potentially important modifiable risk factor for dementia. Future research should focus on more carefully establishing the mechanism by which this occurs, whether the effect is reversed by medication cessation and whether specific anticholinergic medication or classes of medication confer the greatest risk and among which subgroups of the population.

## Supplementary information

**Additional file 1.** Baseline use and 2-year patterns of use of benzodiazepines and anticholinergics of participants with 10-year follow-up (unweighted percentage).

**Additional file 2: Table S1.** a Use of benzodiazepines/Z-drugs at 10-year follow-up stratified by pattern of benzodiazepine/Z-drug use from Y0 to Y2 (percentages are unweighted). b Use of anticholinergics with score of 3 at Y10 stratified by pattern of anticholinergic use from Y0 to Y2 (percentages are unweighted).

**Additional file 3.** Incidence risk ratios for benzodiazepine and anticholinergic medication use and dementia, weighted for attrition and stratified by MMSE, sex and age.

**Additional file 4.** Adjusted incidence rate ratios (95% CI) for dementia from the sensitivity analyses of the main findings using fewer covariates, different dementia exclusion criteria, or different attrition weights.

## Data Availability

Data can be shared through application. For further information please refer to the application forms on the website http://www.cfas.ac.uk/cfas-i/data/#cfasi-data-request

## Abbreviations

ACB: Anticholinergic cognitive burden
ACB12: Anticholinergics with score 1 or 2
ACB3: Anticholinergics with score 3
AGECAT: Automated geriatric examination for computer assisted taxonomy
aIRR: Adjusted incidence rate ratio
BZD: Benzodiazepine or non-benzodiazepine derivatives (Z-drugs)
CI: Confidence interval
IRR: Incidence rate ratio
MMSE: Mini mental state examination
MRC CFAS: Medical research council cognitive function and ageing study
