# Supplementary material for: Anticholinergic and benzodiazepine medication use and risk of incident dementia: a UK cohort study
Source: BMC Geriatr. 2019 Oct 21;19:276. doi: 10.1186/s12877-019-1280-2 (PMC6802337; doi:10.1186/s12877-019-1280-2)
Supplement: Supplementary file 1 — Additional file 1. Baseline use and 2-year patterns of use of benzodiazepines and anticholinergics of participants with 10-year follow-up (unweighted percentage). [file 12877_2019_1280_MOESM1_ESM.docx]

**Additional file 1.** Baseline use and 2-year patterns of use of benzodiazepines and anticholinergics of participants with 10-year follow-up (unweighted percentage).

| Exposure | Pattern of use | Number (%) |
| --- | --- | --- |
| Benzodiazepines (BZD) | None | 2,818 (92.6) |
|  | Any | 227 (7. 5) |
|  | New | 48 (1.6) |
|  | Discontinuing | 57 (1.9) |
|  | Recurrent | 122 (4.0) |
|  | Hypnotic | 166 (5.5) |
|  | Anxiolytic | 44 (1.5) |
|  | Both (Hypnotic and Anxiolytic) | 14 (0.5) |
|  | Short duration | 113 (3.7) |
|  | Long duration | 96 (3.2) |
|  | Both (Short and Long) | 16 (0.5) |
|  | Temazepam | 106 (3.6) |
|  | Nitrazepam | 67 (2.3) |
|  | Diazepam | 33 (1.2) |
|  | Zopiclone | 12 (0.4) |
| Anticholinergics score of 3 (ACB3) | None | 2,875 (94.4) |
|  | Any | 170 (5.6) |
|  | New | 60 (2.0) |
|  | Discontinuing | 40 (1.3) |
|  | Recurrent | 70 (2.3) |
|  | Not antidepressants | 53 (1.7) |
|  | Antidepressants | 106 (3.5) |
|  | Both | 11 (0.4) |
|  | Urologicals | 21 (0.7) |
|  | Gastrointestinals | 19 (0.6) |
|  | Antipsychotics | 15 (0.5) |
|  | Parkinsonian | 3 (0.1) |
|  | Respiratory | 10 (0.3) |
|  | Amitriptyline | 38 (1.3) |
|  | Dosulepin | 37 (1.3) |
| Anticholinergics score of 1 or 2 (ABC12) | None | 1,438 (47.2) |
|  | Any | 1,607 (52.8) |
|  | New | 355 (11.7) |
|  | Discontinuing | 220 (7.2) |
|  | Recurrent | 1,032 (33.9) |
