# Supplementary material for: Anticholinergic and benzodiazepine medication use and risk of incident dementia: a UK cohort study
Source: BMC Geriatr. 2019 Oct 21;19:276. doi: 10.1186/s12877-019-1280-2 (PMC6802337; doi:10.1186/s12877-019-1280-2)
Supplement: Supplementary file 2 — Additional file 2: Table S1. a Use of benzodiazepines/Z-drugs at 10-year follow-up stratified by pattern of benzodiazepine/Z-drug use from Y0 to Y2 (percentages are unweighted). b Use of anticholinergics with score of 3 at Y10 stratified by pattern of anticholinergic use from Y0 to Y2 (percentages are unweighted). [file 12877_2019_1280_MOESM2_ESM.docx]

**Additional file 2.**

**Table a.** Use of benzodiazepines/Z-drugs at 10-year follow-up stratified by pattern of benzodiazepine/Z-drug use from Y0 to Y2 (percentages are unweighted)

**Table b.** Use of anticholinergics with score of 3 at Y10 stratified by pattern of anticholinergic use from Y0 to Y2 (percentages are unweighted)

|  | **Y10 BZD use (%)** |  |  |
| --- | --- | --- | --- |
| Y0/Y2 BZD use | No BZD use | BZD use | Total |
| None | 2674 (96.6) | 93 (3.4) | 2767 (100) |
| New | 18 (36) | 32 (64.0) | 50 (100) |
| Discontinuing | 35 (61.4) | 22 (38.6) | 57 (100) |
| Recurrent | 46 (38.3) | 74 (61.7) | 120 (100) |
| Total | 2773 (92.6) | 221 (7.4) | 2994 (100) |
|  | **Y10 ACB3 use (%)** |  |  |
| Y0/Y2 ACB3 use | No ACB3 use | ACB3 use | Total |
| None | 2633 (93.3) | 190 (6.7) | 2823 (100) |
| New | 36 (59.0) | 25 (41.0) | 61 (100) |
| Discontinuing | 24 (60.0) | 16 (40.0) | 40 (100) |
| Recurrent | 23 (32.9) | 47 (67.1) | 70 (100) |
| Total | 2716 (90.7) | 278 (9.3) | 2994 (100) |
