# Supplementary material for: Anticholinergic and benzodiazepine medication use and risk of incident dementia: a UK cohort study
Source: BMC Geriatr. 2019 Oct 21;19:276. doi: 10.1186/s12877-019-1280-2 (PMC6802337; doi:10.1186/s12877-019-1280-2)
Supplement: Supplementary file 3 — Additional file 3. Incidence risk ratios for benzodiazepine and anticholinergic medication use and dementia, weighted for attrition and stratified by MMSE, sex and age. [file 12877_2019_1280_MOESM3_ESM.docx]

**Additional file 3.** Incidence risk ratios for benzodiazepine and anticholinergic medication use and dementia, weighted for attrition and stratified by MMSE, sex and age

|  | Subgroup | Drug Use | Dementia cases | Total | Incident dementia (%) | IRR (95% CI) | aIRR (95% CI) |
| --- | --- | --- | --- | --- | --- | --- | --- |
| **Stratified by MMSE at Y2** | | |  |  |  |  |  |
| BZD ever-use | ≤ 25 | NO | 89 | 526 | 20.1 |  |  |
|  |  | YES | 17 | 69 | 29.6 | 1.47 (0.92,2.34) | 1.23 (0.74,2.06) |
|  | > 25 | NO | 106 | 2292 | 5.1 |  |  |
|  |  | YES | 8 | 158 | 5.0 | 1.00 (0.48,2.04) | 0.72 (0.35,1.50) |
| ACB3 ever-use | ≤ 25 | NO | 97 | 549 | 21.3 |  |  |
|  |  | YES | 9 | 46 | 21.8 | 1.02 (0.54,1.94) | 0.94 (0.51,1.73) |
|  | > 25 | NO | 101 | 2326 | 4.7 |  |  |
|  |  | YES | 13 | 124 | 11.1 | 2.35*(1.34,4.10) | 2.28*(1.32,3.92) |
| ACB12 ever-use | ≤ 25 | NO | 39 | 244 | 21.3 |  |  |
|  |  | YES | 67 | 351 | 21.4 | 1.01 (0.70,1.46) | 0.78 (0.54,1.12) |
|  | > 25 | NO | 46 | 1194 | 4.3 |  |  |
|  |  | YES | 68 | 1256 | 5.8 | 1.36 (0.94,1.97) | 0.99 (0.68,1.43) |
| **Stratified by sex** | | |  |  |  |  |  |
| BZD ever-use | Male | NO | 56 | 1164 | 5.9 |  |  |
|  |  | YES | 1 | 43 | 3.3 | 0.56 (0.08,3.88) | 0.29 (0.06,1.31) |
|  | Female | NO | 139 | 1654 | 11.0 |  |  |
|  |  | YES | 24 | 184 | 17.0 | 1.55 (1.01,2.40) | 1.17 (0.77,1.78) |
| ACB3 ever-use | Male | NO | 53 | 1157 | 5.6 |  |  |
|  |  | YES | 4 | 50 | 9.9 | 1.77 (0.65,4.80) | 2.06 (0.78,5.46) |
|  | Female | NO | 145 | 1718 | 11.2 |  |  |
|  |  | YES | 18 | 120 | 17.1 | 1.53 (0.94,2.50) | 1.24 (0.77,2.01) |
| ACB12 ever-use | Male | NO | 23 | 620 | 4.5 |  |  |
|  |  | YES | 34 | 587 | 7.1 | 1.56 (0.91,2.66) | 1.11 (0.66,1.89) |
|  | Female | NO | 62 | 818 | 10.9 |  |  |
|  |  | YES | 101 | 1020 | 12.2 | 1.12 (0.81.1.55) | 0.86 (0.63,1.16) |
| **Stratified by birth cohort** | | |  |  |  |  |  |
| BZD ever-use | 1920-29 | NO | 52 | 1776 | 3.5 |  |  |
|  |  | YES | 6 | 104 | 6.5 | 1.83 (0.79,4.27) | 1.31 (0.52,3.27) |
|  | ≤1919 | NO | 143 | 1042 | 16.5 |  |  |
|  |  | YES | 19 | 123 | 20.0 | 1.21 (0.76,1.92) | 1.06 (0.69,1.61) |
| ACB3 ever-use | 1920-29 | NO | 51 | 1775 | 3.5 |  |  |
|  |  | YES | 7 | 105 | 7.1 | 2.01 (0.91,4.46) | 1.16 (0.45,3.01) |
|  | ≤1919 | NO | 147 | 1100 | 16.3 |  |  |
|  |  | YES | 15 | 65 | 25.6 | 1.57 (0.95,2.60) | 1.27 (0.78,2.09) |
| ACB12 ever-use | 1920-29 | NO | 16 | 944 | 2.0 |  |  |
|  |  | YES | 42 | 936 | 5.5 | 2.80 (1.54,5.12) | 1.57 (0.82,3.00) |
|  | ≤1919 | NO | 69 | 494 | 18.0 |  |  |
|  |  | YES | 93 | 671 | 16.2 | 0.90 (0.66,1.21) | 0.77 (0.58,1.02) |

* p<0.01

Abbreviations: ACB12= drugs scoring 1 or 2 on the Anticholinergic Cognitive Burden scale, ACB3= drugs scoring 3 on the Anticholinergic Cognitive Burden scale, BZD=benzodiazepines or Z-drugs, IRR=Attrition-weighted unadjusted incidence rate ratio; aIRR=Attrition-weighted adjusted incidence rate ratio, MMSE = Mini-Mental State Examination
