# Supplementary material for: Anticholinergic and benzodiazepine medication use and risk of incident dementia: a UK cohort study
Source: BMC Geriatr. 2019 Oct 21;19:276. doi: 10.1186/s12877-019-1280-2 (PMC6802337; doi:10.1186/s12877-019-1280-2)
Supplement: Supplementary file 4 — Additional file 4. Adjusted incidence rate ratios (95% CI) for dementia from the sensitivity analyses of the main findings using fewer covariates, different dementia exclusion criteria, or different attrition weights. [file 12877_2019_1280_MOESM4_ESM.docx]

**Additional file 4. Adjusted incidence rate ratios (95% CI) for dementia from the sensitivity analyses of the main findings using fewer covariates, different dementia exclusion criteria, or different attrition weights**

| Exposure |  | Excluding cognition covariates^a^ | Excluding imputed dementia cases | Weighted for drop-out and mortality | Weighted for mortality | Adding diabetes as covariate^b^ | Adding db, me/en, bp and ha^c^ |
| --- | --- | --- | --- | --- | --- | --- | --- |
| BZD use (vs none) | Any | 1.11 (0.75,1.65) | 1.06 (0.71,1.59) | 1.04 (0.68,1.57) | 1.03 (0.66,1.61) | 1.06 (0.72, 1.57) | 1.07 (0.73, 1.57) |
|  | New | 0.70 (0.28,1.75) | 0.65 (0.26,1.63) | 0.63 (0.24,1.64) | 0.64 (0.24,1.69) | 0.65 (0.27,1.55) | 0.63 (0.26,1.51) |
|  | Discontinuing | 1.16 (0.59,2.26) | 1.07 (0.53,2.17) | 1.13 (0.49,2.59) | 1.20 (0.46,3.16) | 1.06 (0.53,2.13) | 1.02 (0.50,2.08) |
|  | Recurrent | 1.29 (0.79,2.09) | 1.29 (0.77,2.15) | 1.23 (0.73,2.07) | 1.15 (0.67,1.99) | 1.31 (0.80,2.14) | 1.36 (0.85,2.19) |
| ACB3 use (vs none) | Any | 1.55* (1.04,2.32) | 1.26 (0.80,2.00) | 1.31 (0.81,2.11) | 1.45 (0.89, 2.38) | 1.30 (0.83,2.03) | 1.28 (0.82,2.01) |
|  | New | 0.95 (0.39,2.33) | 0.85 (0.32,2.23) | 0.85 (0.35,2.07) | 0.91 (0.39, 2.11) | 0.88 (0.34,2.26) | 0.89 (0.35,2.25) |
|  | Discontinuing | 1.57 (0.81,3.05) | 1.19 (0.53,2.69) | 1.32 (0.55,3.13) | 1.58 (0.62,4.04) | 1.20 (0.54,2.71) | 1.18 (0.53,2.64) |
|  | Recurrent | 2.02** (1.21,3.39) | 1.66 (0.97,2.85) | 1.64 (0.94,2.86) | 1.74 (0.98,3.09) | 1.71 (1.01,2.88)* | 1.68 (0.99,2.86) |
| ACB12 (vs none) | Any use | 0.97 (0.74,1.26) | 0.89 (0.68,1.17) | 0.86 (0.65,1.14) | 0.92 (0.69,1.24) | 0.89 (0.68,1.17) | 0.89 (0.67,1.19) |

* p<0.05 ** p<0.01

a Adjusted for all covariates except for MMSE at Y2, change in MMSE (Y0 to Y2), MMSE orientation sub-score at Y2, disability, and arm of the study.

b Adjusted for all covariates + diagnosed diabetes in Y0 or Y2, weighted for refusal and move

c Adjusted for all covariates + diagnosed diabetes, having blood pressure, suffering meningitis/encephalitis and suffering from heart attack in Y0 or Y2, weighted for refusal and move
